# 3-Amino-1-(4-fluoro­phen­yl)-7-meth­oxy-1*H*-benzo[*f*]chromene-2-carbonitrile

**DOI:** 10.1107/S1600536813005473

**Published:** 2013-03-02

**Authors:** Abd El-Galil E. Amr, Ahmed M. El-Agrody, Mohamed A. Al-Omar, Seik Weng Ng, Edward R. T. Tiekink

**Affiliations:** a Drug Exploration & Development Chair (DEDC), College of Pharmacy, King Saud University, Riyadh 11451, Saudi Arabia; b Applied Organic Chemistry Department, National Research Center, Dokki 12622, Cairo, Egypt; c Chemistry Department, Faculty of Science, King Khalid University, Abha 61413, PO Box 9004, Saudi Arabia; d Chemistry Department, Faculty of Science, Al-Azhar University, Nasr City, Cairo, 11884, Egypt; e Pharmaceutical Chemistry Department, College of Pharmacy, King Saud University, Riyadh 11451, Saudi Arabia; f Department of Chemistry, University of Malaya, 50603 Kuala Lumpur, Malaysia; g Chemistry Department, Faculty of Science, King Abdulaziz University, PO Box 80203 Jeddah, Saudi Arabia

## Abstract

In the title compound, C_21_H_15_FN_2_O_2_, the furan ring has a flattened half-chair conformation [the methine C atom lies 0.136 (2) Å above the C_5_ plane which has an r.m.s. deviation of 0.0229 Å]. Overall, the 1*H*-benzo[*f*]chromene fused-ring system approximates a plane (r.m.s. deviation of the 14 non-H atoms = 0.049 Å). The fluoro­benzene ring is almost perpendicular to this plane [dihedral angle = 89.58 (8)°]. Zigzag supra­molecular tapes along the *b* axis are the most notable feature of the crystal packing. This arises through an alternating sequence of 12-membered {⋯HNC_3_N}_2_ and eight-membered {⋯HNCO}_2_ synthons. These are connected into a three-dimensional architecture by π–π [inter­centroid distance for centrosymmetrically related fluoro­benzene rings = 3.5181 (10) Å] and C—H⋯π inter­actions.

## Related literature

For a related structure and background to 4*H*-chromene derivatives, see: El-Agrody *et al.* (2013 ▶). For related structures, see: Wang *et al.* (2008 ▶); Shekhar *et al.* (2012 ▶);
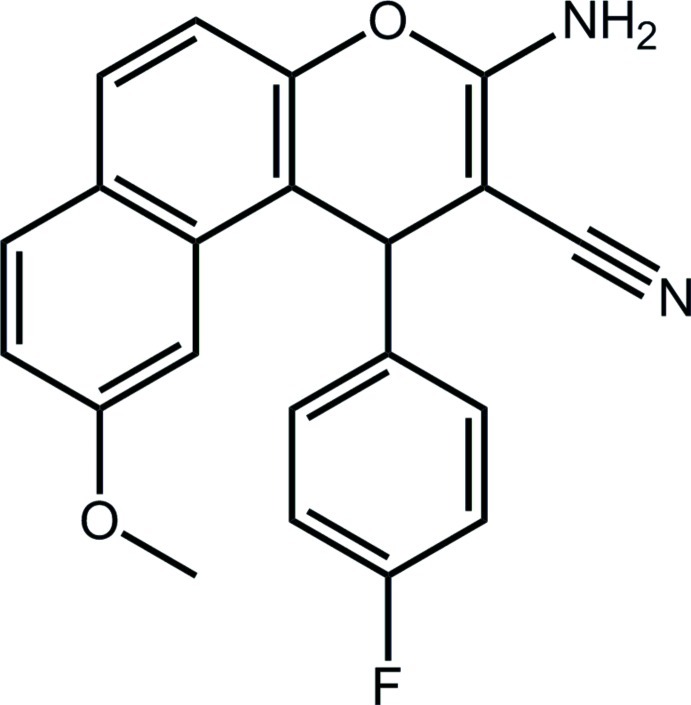


## Experimental

### 

#### Crystal data

C_21_H_15_FN_2_O_2_
*M* *_r_* = 346.35
Triclinic, 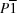

*a* = 8.7798 (9) Å
*b* = 9.6329 (6) Å
*c* = 10.9130 (11) Å
α = 77.074 (7)°
β = 68.414 (10)°
γ = 87.083 (7)°
*V* = 835.99 (13) Å^3^
*Z* = 2
Mo *K*α radiation
μ = 0.10 mm^−1^
*T* = 295 K
0.30 × 0.30 × 0.10 mm

#### Data collection

Agilent SuperNova Dual diffractometer with an Atlas detector
Absorption correction: multi-scan (*CrysAlis PRO*; Agilent, 2011 ▶) *T*_min_ = 0.821, *T*_max_ = 1.000
7587 measured reflections
3868 independent reflections
2569 reflections with *I* > 2σ(*I*)
*R*_int_ = 0.029

#### Refinement

*R*[*F* ^2^ > 2σ(*F* ^2^)] = 0.052
*wR*(*F* ^2^) = 0.145
*S* = 1.02
3868 reflections
244 parameters
H atoms treated by a mixture of independent and constrained refinement
Δρ_max_ = 0.19 e Å^−3^
Δρ_min_ = −0.16 e Å^−3^

### 

Data collection: *CrysAlis PRO* (Agilent, 2011 ▶); cell refinement: *CrysAlis PRO*; data reduction: *CrysAlis PRO*; program(s) used to solve structure: *SHELXS97* (Sheldrick, 2008 ▶); program(s) used to refine structure: *SHELXL97* (Sheldrick, 2008 ▶); molecular graphics: *ORTEP-3 for Windows* (Farrugia, 2012 ▶) and *DIAMOND* (Brandenburg, 2006 ▶); software used to prepare material for publication: *publCIF* (Westrip, 2010 ▶).

## Supplementary Material

Crystal structure: contains datablock(s) global, I. DOI: 10.1107/S1600536813005473/hb7049sup1.cif

Structure factors: contains datablock(s) I. DOI: 10.1107/S1600536813005473/hb7049Isup2.hkl

Supplementary material file. DOI: 10.1107/S1600536813005473/hb7049Isup3.cml

Additional supplementary materials:  crystallographic information; 3D view; checkCIF report

## Figures and Tables

**Table 1:** Hydrogen-bond geometry (Å, °) *Cg*1 is the centroid of the C15–CC20 ring.

*D*—H⋯*A*	*D*—H	H⋯*A*	*D*⋯*A*	*D*—H⋯*A*
N1—H1⋯N2^i^	0.89 (2)	2.34 (3)	3.189 (2)	160 (2)
N1—H2⋯O1^ii^	0.87 (2)	2.36 (3)	3.219 (2)	169 (2)
C19—H19⋯*Cg*1^iii^	0.93	2.90	3.831 (2)	174

Symmetry codes: (i) 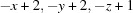; (ii) 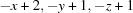; (iii) 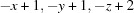.

**Fig. 1.**
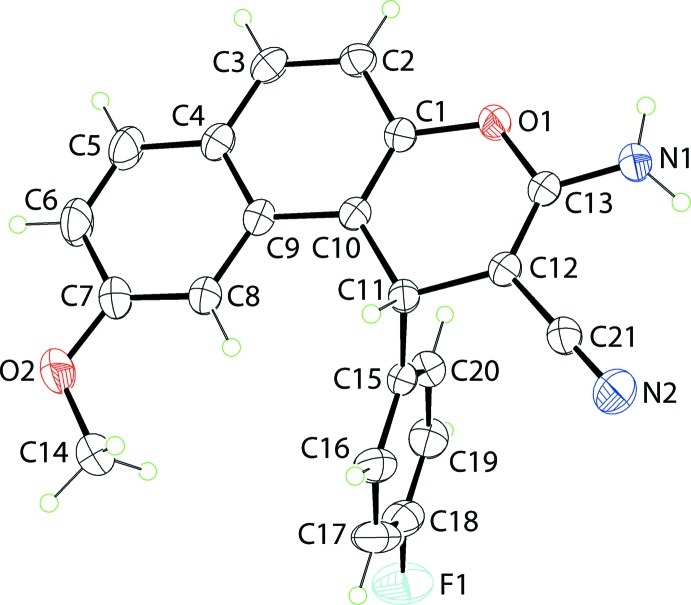
The molecular structure of (I) showing displacement ellipsoids at the 35% probability level.

**Fig. 2.**
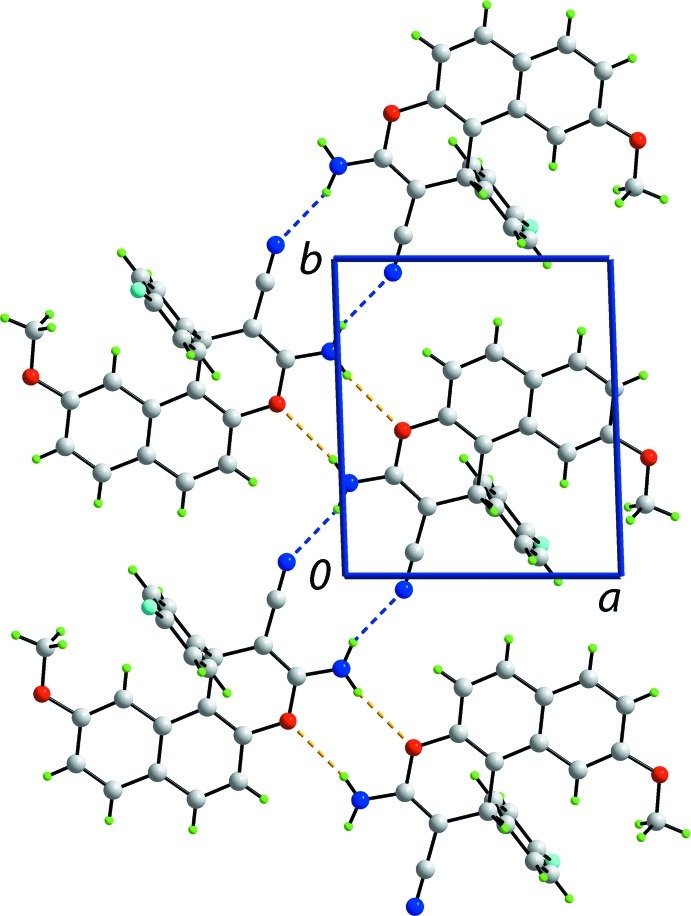
A view of the supramolecular zigzag tape along the b axis in (I). The N—H···N and N—H···O hydrogen bonds are shown as blue and orange dashed lines, respectively.

**Fig. 3.**
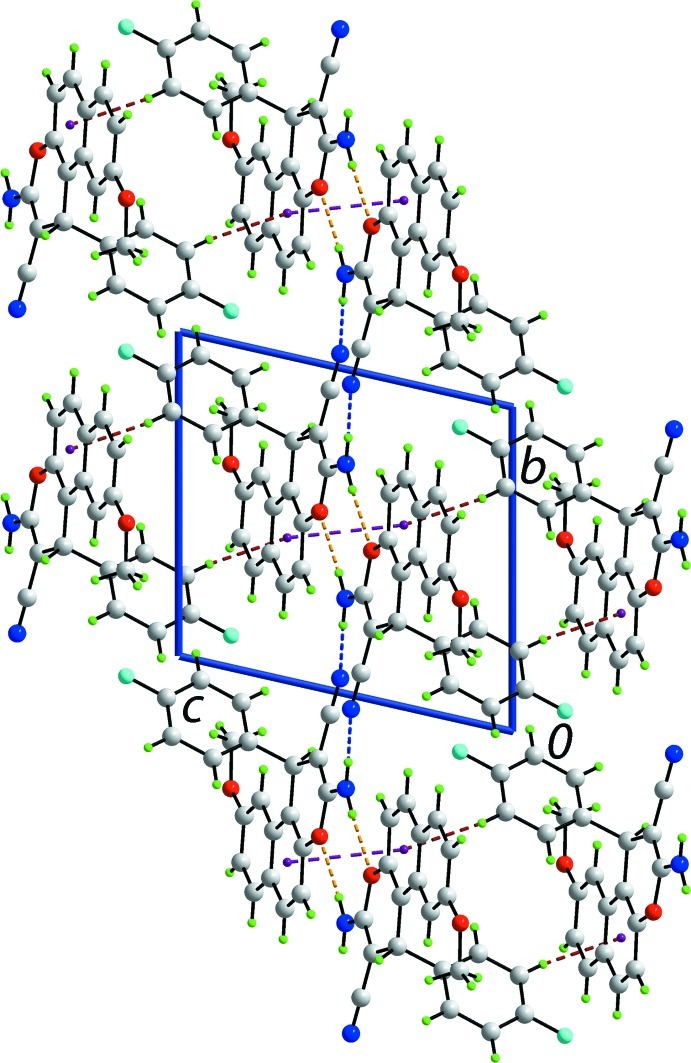
A view in projection down the a axis of the crystal packing in (I). The N—H···N, N—H···O, C—H···π and π—π interactions are shown as blue, orange, brown and purple dashed lines, respectively.

## supplementary crystallographic information

### Comment

As part of our ongoing studies of 4*H*-Chromene derivatives (El-Agrody *et al.*, 2013), the crystal and molecular structure of the title compound, (I), is described herein.

In (I), Fig. 1, the furan ring has a flattened half-chair conformation with the methine-C11 atom lying only 0.136 (2) Å above the plane of the remaining atoms (r.m.s. deviation = 0.0229 Å). In fact, the 14 non-hydrogen atoms of the 1*H*-benzo[*f*]chromene fused-ring system are co-planar with a r.m.s. deviation of the fitted atoms = 0.049 Å. The maximum deviations are 0.068 (2) Å for the aforementioned methine-C11 atom and -0.107 (2) Å for the adjacent C12 atom. The fluorobenzene ring is perpendicular to the 1*H*-benzo[*f*]chromene residue, forming a dihedral angle of 89.58 (8)°. The methoxy group is co-planar with the ring to which it is attached as manifested in the C14—O2—C7—C6 torsion angle of 177.17 (19)°. The structure described here resembles very closely those of the 4-bromo (Wang *et al.*, 2008) and 2-CF_3_ (Shekhar *et al.*, 2012) derivatives of the parent compound, as well as that of the 8-methoxy analogue (El-Agrody *et al.*, 2013).

In the crystal packing, zigzag tapes are formed along the *b* axis *via* an alternating sequence of 12-membered {···HNC_3_N}_2_, arising from amine-*N*—*H*···*N*(cyano) hydrogen bonds, and eight-membered {···HNCO}_2_, arising from amine-*N*—*H*···*O*(furan) hydrogen bonds, synthons, Fig. 2 and Table 1. These are connected into a layer in the *ab* plane by π—π interactions occurring between centrosymmetrically related fluorobenzene rings [inter-centroid distance = 3.5181 (10) Å for symmetry operation: 1 - *x*, 1 - *y*, 1 - *z*]. Layers are connected along the *c* axis by C—H···π interactions where both the donor atom and acceptor-π system are derived from fluorobenzene rings, Fig. 3 and Table 1, highlighting the key role this residue plays in consolidating the crystal structure of (I).

### Experimental

A solution of 7-methoxy-2-naphthol (0.01 mol) in EtOH (30 ml) was treated with α-cyano-*p*-fluorocinnamonitrile (0.01 mol) and piperidine (0.5 ml). The reaction mixture was heated until complete precipitation occurred (reaction time: 60 min). The solid product which formed was collected by filtration and recrystallized from ethanol to give the title compound, (I), as light-brown prisms; *M*.pt: 533–534 K.

### Refinement

The C-bound H atoms were geometrically placed (C–H = 0.93–0.98 Å) and refined as riding with *U_iso_*(H) = 1.2*U_eq_*(C). The N-bound-H atoms were refined freely.

### Crystal data

**Table:**

C_21_H_15_FN_2_O_2_	*Z* = 2
*M**_r_* = 346.35	*F*(000) = 360
Triclinic, *P*1	*D*_x_ = 1.376 Mg m^−^^3^
Hall symbol: -P 1	Mo *K*α radiation, λ = 0.71073 Å
*a* = 8.7798 (9) Å	Cell parameters from 1958 reflections
*b* = 9.6329 (6) Å	θ = 3.1–27.5°
*c* = 10.9130 (11) Å	µ = 0.10 mm^−^^1^
α = 77.074 (7)°	*T* = 295 K
β = 68.414 (10)°	Prism, light-brown
γ = 87.083 (7)°	0.30 × 0.30 × 0.10 mm
*V* = 835.99 (13) Å^3^	

### Data collection

**Table:**

Agilent SuperNova Dual diffractometer with an Atlas detector	3868 independent reflections
Radiation source: SuperNova (Mo) X-ray Source	2569 reflections with *I* > 2σ(*I*)
Mirror monochromator	*R*_int_ = 0.029
Detector resolution: 10.4041 pixels mm^-1^	θ_max_ = 27.6°, θ_min_ = 3.1°
ω scan	*h* = −8→11
Absorption correction: multi-scan (*CrysAlis PRO*; Agilent, 2011)	*k* = −12→12
*T*_min_ = 0.821, *T*_max_ = 1.000	*l* = −13→14
7587 measured reflections	

### Refinement

**Table:**

Refinement on *F*^2^	Secondary atom site location: difference Fourier map
Least-squares matrix: full	Hydrogen site location: inferred from neighbouring sites
*R*[*F*^2^ > 2σ(*F*^2^)] = 0.052	H atoms treated by a mixture of independent and constrained refinement
*wR*(*F*^2^) = 0.145	*w* = 1/[σ^2^(*F*_o_^2^) + (0.0577*P*)^2^ + 0.1106*P*] where *P* = (*F*_o_^2^ + 2*F*_c_^2^)/3
*S* = 1.02	(Δ/σ)_max_ < 0.001
3868 reflections	Δρ_max_ = 0.19 e Å^−^^3^
244 parameters	Δρ_min_ = −0.16 e Å^−^^3^
0 restraints	Extinction correction: *SHELXL97* (Sheldrick, 2008), Fc^*^=kFc[1+0.001xFc^2^λ^3^/sin(2θ)]^-1/4^
Primary atom site location: structure-invariant direct methods	Extinction coefficient: 0.011 (3)

### Special details

**Table:**

Geometry. All e.s.d.'s (except the e.s.d. in the dihedral angle between two l.s. planes) are estimated using the full covariance matrix. The cell e.s.d.'s are taken into account individually in the estimation of e.s.d.'s in distances, angles and torsion angles; correlations between e.s.d.'s in cell parameters are only used when they are defined by crystal symmetry. An approximate (isotropic) treatment of cell e.s.d.'s is used for estimating e.s.d.'s involving l.s. planes.
Refinement. Refinement of *F*^2^ against ALL reflections. The weighted *R*-factor *wR* and goodness of fit *S* are based on *F*^2^, conventional *R*-factors *R* are based on *F*, with *F* set to zero for negative *F*^2^. The threshold expression of *F*^2^ > σ(*F*^2^) is used only for calculating *R*-factors(gt) *etc*. and is not relevant to the choice of reflections for refinement. *R*-factors based on *F*^2^ are statistically about twice as large as those based on *F*, and *R*- factors based on ALL data will be even larger.

### Fractional atomic coordinates and isotropic or equivalent isotropic displacement parameters (Å^2^)

**Table:**

	*x*	*y*	*z*	*U*_iso_*/*U*_eq_	
F1	0.2857 (2)	0.90095 (14)	1.15726 (13)	0.0943 (5)	
O1	0.77412 (14)	0.54184 (11)	0.57793 (13)	0.0452 (3)	
O2	−0.11449 (16)	0.62729 (15)	0.84385 (16)	0.0692 (5)	
N1	0.97391 (19)	0.70744 (19)	0.49939 (18)	0.0537 (5)	
H1	1.020 (3)	0.790 (2)	0.492 (2)	0.078 (7)*	
H2	1.030 (3)	0.633 (3)	0.480 (2)	0.080 (7)*	
N2	0.7893 (2)	1.04350 (16)	0.51621 (19)	0.0677 (6)	
C1	0.6096 (2)	0.49698 (16)	0.62727 (16)	0.0366 (4)	
C2	0.5869 (2)	0.35217 (16)	0.63298 (17)	0.0414 (4)	
H2A	0.6766	0.2958	0.6040	0.050*	
C3	0.4316 (2)	0.29574 (17)	0.68165 (17)	0.0446 (4)	
H3	0.4156	0.1996	0.6869	0.054*	
C4	0.2945 (2)	0.38016 (17)	0.72431 (17)	0.0414 (4)	
C5	0.1313 (3)	0.3245 (2)	0.7744 (2)	0.0564 (5)	
H5	0.1133	0.2285	0.7807	0.068*	
C6	0.0011 (3)	0.4075 (2)	0.8134 (2)	0.0633 (6)	
H6	−0.1047	0.3681	0.8469	0.076*	
C7	0.0254 (2)	0.5534 (2)	0.8034 (2)	0.0513 (5)	
C8	0.1808 (2)	0.61196 (18)	0.75438 (18)	0.0441 (4)	
H8	0.1957	0.7089	0.7465	0.053*	
C9	0.3190 (2)	0.52689 (16)	0.71562 (16)	0.0372 (4)	
C10	0.4828 (2)	0.58616 (15)	0.66581 (15)	0.0343 (4)	
C11	0.51257 (19)	0.74259 (15)	0.65506 (16)	0.0351 (4)	
H11	0.4541	0.7977	0.6002	0.042*	
C12	0.6944 (2)	0.78082 (15)	0.58154 (16)	0.0373 (4)	
C13	0.8109 (2)	0.68330 (16)	0.55414 (17)	0.0392 (4)	
C14	−0.0958 (3)	0.7740 (2)	0.8413 (3)	0.0734 (7)	
H14A	−0.2018	0.8130	0.8766	0.110*	
H14B	−0.0391	0.8247	0.7500	0.110*	
H14C	−0.0338	0.7832	0.8957	0.110*	
C15	0.44924 (19)	0.78420 (15)	0.79176 (16)	0.0370 (4)	
C16	0.3506 (2)	0.89994 (17)	0.8121 (2)	0.0509 (5)	
H16	0.3206	0.9516	0.7421	0.061*	
C17	0.2955 (3)	0.9403 (2)	0.9351 (2)	0.0634 (6)	
H17	0.2295	1.0183	0.9483	0.076*	
C18	0.3407 (3)	0.8624 (2)	1.0362 (2)	0.0575 (6)	
C19	0.4377 (3)	0.7473 (2)	1.02070 (19)	0.0548 (5)	
H19	0.4670	0.6963	1.0913	0.066*	
C20	0.4910 (2)	0.70854 (18)	0.89766 (17)	0.0454 (4)	
H20	0.5564	0.6299	0.8858	0.054*	
C21	0.7466 (2)	0.92564 (17)	0.54616 (18)	0.0441 (4)	

### Atomic displacement parameters (Å^2^)

**Table:**

	*U*^11^	*U*^22^	*U*^33^	*U*^12^	*U*^13^	*U*^23^
F1	0.1272 (14)	0.0935 (10)	0.0578 (8)	0.0132 (9)	−0.0173 (8)	−0.0393 (7)
O1	0.0329 (7)	0.0349 (6)	0.0654 (8)	0.0018 (5)	−0.0135 (6)	−0.0140 (5)
O2	0.0339 (7)	0.0678 (9)	0.1011 (12)	−0.0024 (6)	−0.0146 (8)	−0.0258 (8)
N1	0.0325 (9)	0.0453 (9)	0.0791 (12)	−0.0011 (7)	−0.0103 (8)	−0.0220 (8)
N2	0.0572 (11)	0.0378 (9)	0.0939 (14)	−0.0085 (7)	−0.0128 (10)	−0.0095 (8)
C1	0.0352 (9)	0.0358 (8)	0.0379 (9)	−0.0014 (6)	−0.0123 (7)	−0.0075 (6)
C2	0.0444 (10)	0.0335 (8)	0.0458 (10)	0.0038 (7)	−0.0160 (8)	−0.0093 (7)
C3	0.0556 (12)	0.0310 (8)	0.0483 (10)	−0.0068 (7)	−0.0209 (9)	−0.0059 (7)
C4	0.0446 (10)	0.0375 (9)	0.0418 (9)	−0.0071 (7)	−0.0171 (8)	−0.0043 (7)
C5	0.0511 (12)	0.0434 (10)	0.0715 (13)	−0.0151 (8)	−0.0205 (10)	−0.0060 (9)
C6	0.0401 (11)	0.0596 (12)	0.0834 (15)	−0.0168 (9)	−0.0165 (11)	−0.0085 (10)
C7	0.0355 (10)	0.0569 (11)	0.0599 (12)	−0.0041 (8)	−0.0149 (9)	−0.0125 (9)
C8	0.0373 (10)	0.0430 (9)	0.0520 (11)	−0.0034 (7)	−0.0156 (8)	−0.0107 (7)
C9	0.0376 (9)	0.0387 (9)	0.0361 (9)	−0.0049 (7)	−0.0151 (7)	−0.0060 (6)
C10	0.0355 (9)	0.0332 (8)	0.0346 (8)	−0.0020 (6)	−0.0130 (7)	−0.0074 (6)
C11	0.0303 (8)	0.0328 (8)	0.0418 (9)	0.0004 (6)	−0.0130 (7)	−0.0076 (6)
C12	0.0353 (9)	0.0321 (8)	0.0420 (9)	−0.0023 (6)	−0.0114 (7)	−0.0076 (7)
C13	0.0361 (9)	0.0343 (8)	0.0459 (10)	−0.0027 (7)	−0.0122 (8)	−0.0104 (7)
C14	0.0436 (12)	0.0753 (15)	0.1080 (19)	0.0078 (10)	−0.0225 (12)	−0.0435 (13)
C15	0.0321 (8)	0.0325 (8)	0.0435 (9)	−0.0049 (6)	−0.0092 (7)	−0.0095 (7)
C16	0.0594 (12)	0.0367 (9)	0.0555 (11)	0.0048 (8)	−0.0190 (9)	−0.0122 (8)
C17	0.0748 (16)	0.0451 (11)	0.0654 (14)	0.0113 (9)	−0.0140 (11)	−0.0251 (9)
C18	0.0646 (14)	0.0588 (12)	0.0443 (11)	−0.0064 (10)	−0.0069 (10)	−0.0220 (9)
C19	0.0575 (13)	0.0618 (12)	0.0440 (11)	−0.0024 (9)	−0.0169 (9)	−0.0110 (9)
C20	0.0409 (10)	0.0473 (10)	0.0469 (10)	0.0013 (7)	−0.0142 (8)	−0.0115 (8)
C21	0.0345 (9)	0.0397 (9)	0.0531 (10)	−0.0008 (7)	−0.0099 (8)	−0.0106 (7)

### Geometric parameters (Å, º)

**Table:**

F1—C18	1.359 (2)	C8—C9	1.414 (2)
O1—C13	1.3613 (18)	C8—H8	0.9300
O1—C1	1.3966 (19)	C9—C10	1.434 (2)
O2—C7	1.365 (2)	C10—C11	1.514 (2)
O2—C14	1.424 (2)	C11—C12	1.521 (2)
N1—C13	1.343 (2)	C11—C15	1.526 (2)
N1—H1	0.89 (2)	C11—H11	0.9800
N1—H2	0.87 (2)	C12—C13	1.349 (2)
N2—C21	1.151 (2)	C12—C21	1.414 (2)
C1—C10	1.369 (2)	C14—H14A	0.9600
C1—C2	1.403 (2)	C14—H14B	0.9600
C2—C3	1.360 (2)	C14—H14C	0.9600
C2—H2A	0.9300	C15—C20	1.382 (2)
C3—C4	1.409 (3)	C15—C16	1.383 (2)
C3—H3	0.9300	C16—C17	1.387 (3)
C4—C5	1.418 (2)	C16—H16	0.9300
C4—C9	1.417 (2)	C17—C18	1.363 (3)
C5—C6	1.352 (3)	C17—H17	0.9300
C5—H5	0.9300	C18—C19	1.366 (3)
C6—C7	1.405 (3)	C19—C20	1.380 (2)
C6—H6	0.9300	C19—H19	0.9300
C7—C8	1.369 (2)	C20—H20	0.9300
			
C13—O1—C1	118.63 (12)	C10—C11—C15	113.09 (12)
C7—O2—C14	117.06 (15)	C12—C11—C15	110.77 (13)
C13—N1—H1	122.0 (15)	C10—C11—H11	107.8
C13—N1—H2	114.6 (16)	C12—C11—H11	107.8
H1—N1—H2	123 (2)	C15—C11—H11	107.8
C10—C1—O1	123.09 (14)	C13—C12—C21	117.59 (15)
C10—C1—C2	123.33 (16)	C13—C12—C11	123.60 (13)
O1—C1—C2	113.58 (14)	C21—C12—C11	118.71 (14)
C3—C2—C1	118.86 (16)	N1—C13—C12	127.20 (15)
C3—C2—H2A	120.6	N1—C13—O1	110.29 (14)
C1—C2—H2A	120.6	C12—C13—O1	122.50 (15)
C2—C3—C4	121.27 (15)	O2—C14—H14A	109.5
C2—C3—H3	119.4	O2—C14—H14B	109.5
C4—C3—H3	119.4	H14A—C14—H14B	109.5
C3—C4—C5	122.50 (16)	O2—C14—H14C	109.5
C3—C4—C9	119.31 (16)	H14A—C14—H14C	109.5
C5—C4—C9	118.18 (17)	H14B—C14—H14C	109.5
C6—C5—C4	121.72 (17)	C20—C15—C16	118.34 (16)
C6—C5—H5	119.1	C20—C15—C11	120.85 (15)
C4—C5—H5	119.1	C16—C15—C11	120.80 (16)
C5—C6—C7	120.09 (18)	C15—C16—C17	121.12 (19)
C5—C6—H6	120.0	C15—C16—H16	119.4
C7—C6—H6	120.0	C17—C16—H16	119.4
O2—C7—C8	124.64 (17)	C18—C17—C16	118.26 (18)
O2—C7—C6	115.14 (17)	C18—C17—H17	120.9
C8—C7—C6	120.21 (18)	C16—C17—H17	120.9
C7—C8—C9	120.80 (16)	F1—C18—C19	119.1 (2)
C7—C8—H8	119.6	F1—C18—C17	118.35 (19)
C9—C8—H8	119.6	C19—C18—C17	122.58 (18)
C8—C9—C4	118.98 (15)	C18—C19—C20	118.36 (19)
C8—C9—C10	121.54 (15)	C18—C19—H19	120.8
C4—C9—C10	119.47 (15)	C20—C19—H19	120.8
C1—C10—C9	117.74 (14)	C15—C20—C19	121.33 (17)
C1—C10—C11	121.65 (14)	C15—C20—H20	119.3
C9—C10—C11	120.61 (14)	C19—C20—H20	119.3
C10—C11—C12	109.49 (13)	N2—C21—C12	179.4 (2)
			
C13—O1—C1—C10	−6.8 (2)	C4—C9—C10—C11	179.82 (14)
C13—O1—C1—C2	172.96 (14)	C1—C10—C11—C12	7.1 (2)
C10—C1—C2—C3	−1.4 (3)	C9—C10—C11—C12	−172.08 (14)
O1—C1—C2—C3	178.88 (15)	C1—C10—C11—C15	−116.92 (17)
C1—C2—C3—C4	0.8 (3)	C9—C10—C11—C15	63.9 (2)
C2—C3—C4—C5	179.49 (17)	C10—C11—C12—C13	−11.2 (2)
C2—C3—C4—C9	0.3 (3)	C15—C11—C12—C13	114.20 (18)
C3—C4—C5—C6	−179.2 (2)	C10—C11—C12—C21	172.48 (15)
C9—C4—C5—C6	0.0 (3)	C15—C11—C12—C21	−62.1 (2)
C4—C5—C6—C7	0.7 (4)	C21—C12—C13—N1	1.9 (3)
C14—O2—C7—C8	−3.6 (3)	C11—C12—C13—N1	−174.45 (18)
C14—O2—C7—C6	177.17 (19)	C21—C12—C13—O1	−176.76 (15)
C5—C6—C7—O2	179.2 (2)	C11—C12—C13—O1	6.9 (3)
C5—C6—C7—C8	−0.1 (3)	C1—O1—C13—N1	−176.14 (15)
O2—C7—C8—C9	179.56 (18)	C1—O1—C13—C12	2.7 (2)
C6—C7—C8—C9	−1.2 (3)	C10—C11—C15—C20	51.2 (2)
C7—C8—C9—C4	1.9 (3)	C12—C11—C15—C20	−72.18 (18)
C7—C8—C9—C10	−179.13 (17)	C10—C11—C15—C16	−129.82 (17)
C3—C4—C9—C8	177.90 (16)	C12—C11—C15—C16	106.85 (18)
C5—C4—C9—C8	−1.3 (3)	C20—C15—C16—C17	0.5 (3)
C3—C4—C9—C10	−1.1 (2)	C11—C15—C16—C17	−178.57 (17)
C5—C4—C9—C10	179.75 (16)	C15—C16—C17—C18	−0.2 (3)
O1—C1—C10—C9	−179.64 (14)	C16—C17—C18—F1	−179.39 (18)
C2—C1—C10—C9	0.6 (3)	C16—C17—C18—C19	0.1 (3)
O1—C1—C10—C11	1.1 (3)	F1—C18—C19—C20	179.25 (17)
C2—C1—C10—C11	−178.59 (15)	C17—C18—C19—C20	−0.2 (3)
C8—C9—C10—C1	−178.36 (16)	C16—C15—C20—C19	−0.6 (3)
C4—C9—C10—C1	0.6 (2)	C11—C15—C20—C19	178.43 (16)
C8—C9—C10—C11	0.9 (3)	C18—C19—C20—C15	0.5 (3)

### Hydrogen-bond geometry (Å, º)

Cg1 is the centroid of the C15–CC20 ring.

**Table:**

*D*—H···*A*	*D*—H	H···*A*	*D*···*A*	*D*—H···*A*
N1—H1···N2^i^	0.89 (2)	2.34 (3)	3.189 (2)	160 (2)
N1—H2···O1^ii^	0.87 (2)	2.36 (3)	3.219 (2)	169 (2)
C19—H19···*Cg*1^iii^	0.93	2.90	3.831 (2)	174

Symmetry codes: (i) −*x*+2, −*y*+2, −*z*+1; (ii) −*x*+2, −*y*+1, −*z*+1; (iii) −*x*+1, −*y*+1, −*z*+2.
